# Bodily emotional expressions are a primary source of information for dogs, but not for humans

**DOI:** 10.1007/s10071-021-01471-x

**Published:** 2021-01-28

**Authors:** Catia Correia-Caeiro, Kun Guo, Daniel Mills

**Affiliations:** 1 School of Psychology, University of Lincoln, Lincoln, UK; 2 School of Life Sciences, University of Lincoln, Lincoln, UK; 3 Primate Research Institute, Kyoto University, Inuyama, Japan

**Keywords:** Dogs, Humans, Emotion, Face and body, Comparative perception

## Abstract

Dogs have remarkable abilities to synergise their behaviour with that of people, but how dogs read facial and bodily emotional cues in comparison to humans remains unclear. Both species share the same ecological niche, are highly social and expressive, making them an ideal comparative model for intra- and inter-species emotion perception. We compared eye-tracking data from unrestrained humans and dogs when viewing dynamic and naturalistic emotional expressions in humans and dogs. Dogs attended more to the body than the head of human and dog figures, unlike humans who focused more on the head of both species. Dogs and humans also showed a clear age effect that reduced head gaze. Our results indicate a species-specific evolutionary adaptation for emotion perception, which is only partly modified for heterospecific cues. These results have important implications for managing the risk associated with human–dog interactions, where expressive and perceptual differences are crucial.

**Supplementary Information:**

The online version contains supplementary material available at 10.1007/s10071-021-01471-x.

## Introduction

Dogs serve a variety of roles in society (as companion, working and therapy animals) of significant benefit to human physical and mental health and great social and economic value (Hall et al. 2016). There is growing evidence to support an association between aspects of dog ownership and a wide range of emotional, behavioural, cognitive, educational and social benefits (e.g. increased social competence, social networks and social interaction) (Hall et al. 2016). The safeguarding of rewarding human–dog interaction requires timely and appropriate understanding of emotional expressions in both humans and dogs from each other’s perspective. However, we currently know little about how interspecies emotion perception is achieved.

Facial and bodily expressions are generally considered the dominant channels of emotional expression (at least) in humans, and they quickly attract visual attention (Vuilleumier 2005). Humans are extremely sensitive to each other’s facial expressions, as we show inborn predispositions to process expressive facial cues, quickly perfect relevant perceptual capacities (e.g. expression categorisation accuracy) through increasing practice and exposure over time, and exhibit face-specific and emotion-sensitive cognitive and neural processes (Leopold and Rhodes 2010; Schirmer and Adolphs 2017). Additionally, humans tend to display similar stereotypical gaze allocation with longer viewing times at animal faces (Kujala et al. 2012). This face "magnetism" is not restricted to humans. Non-human primates immediately locate the face in pictures of conspecifics and humans, looking more towards heads than bodies (Kano and Tomonaga 2009). Several domestic species, including sheep (Kendrick 2008) and horses (Proops et al. 2018), have also been shown to be able to process conspecific and/or human facial expressions (Tate et al. 2006).

Dogs are also sensitive to human facial expressions and are able to use these facial cues to guide their actions (Merola et al. 2012). They can discriminate smiling from neutral faces (Nagasawa et al. 2011) and happy from disgusted faces (Buttelmann and Tomasello 2013). They also show differential behavioural [e.g. avoiding angry faces and attending more to fearful faces (Deputte and Doll 2011)] and physiological reactions [e.g.  changes in heart rate (Barber et al. 2017)] to emotional faces. Furthermore, dogs can selectively respond to emotional human faces using configural facial cues (Müller et al. 2015) focusing mostly on the eye region, similarly to humans (Somppi et al. 2014). Although dogs can discriminate some prototypical human emotional expressions (e.g. happiness vs. anger) via visual, auditory and olfactory inputs (e.g. Albuquerque et al. 2016; Semin et al. 2019), we currently know little about how interspecies emotion perception is achieved between humans and dogs, such as whether a similar perceptual mechanism is adopted in these two species.

As these studies only focus on facial expressions, they potentially bias our understanding of human–dog emotion perception towards the importance of the face. Although there is a mammalian homology in emotional brain pathways and in facial musculature between humans and dogs, dogs display different patterns of expression-specific facial musculature movements compared to humans in comparable states of emotional arousal (e.g. Mouth stretch—Action Unit 27 (AU27), where dogs open their mouths wide, and Cheek raiser—AU6, where humans contract the muscle around the eyes to pull the cheeks upwards as part of “happy” faces; Caeiro et al. 2017b). When exploring different categories of human and dog facial expressions, human viewers gaze more frequently and for a longer time at the eyes of expressive human faces, but longer at the mouth of expressive dog faces (Guo et al. 2019) or equally long at the eyes and mouth of expressive dog faces (Correia-Caeiro et al. 2020). The lack of commonality in facial expressions and face-viewing gaze allocation between these two species questions the degree to which humans and dogs can appropriately interpret each other’s emotional state based on facial expression alone.

The human body is also a source of important cues (Gelder 2006; Dael et al. 2012b), which can impact emotion recognition (Gelder 2006; Aviezer et al. 2012). Bodies seem to convey certain emotional states more effectively from afar by transmitting larger and more dynamic cues (Dael et al. 2012a; Martinez et al. 2016). Human hand gestures and body postures have been highlighted as having a possible role in human–dog communication (Skyrme and Mills 2010; D’Aniello et al. 2016), but it is still unclear which cues (facial vs. bodily) are most important when reading each other’s emotions in similar real-world situations.

We also do not know how ageing and gender may affect humans’ and dogs’ preference to attend to different emotional cues. Such effects may underpin the gender- and age-dependent understanding of emotions reported in humans (Nummenmaa et al. 2012; Sullivan et al. 2017), and age-dependent risk profiles associated with human–dog interaction (Hsu and Sun 2010; Westgarth et al. 2018).

Therefore, in this study, we compared relative gaze allocation at the face and body regions between humans and dogs in viewing videos of different categories of whole-body human and dog emotional expressions. We aimed to answer the following questions: (1) is human gaze affected by emotion or species observed? (2) Does the viewer's age or sex influence human gaze? (3) Is dog gaze affected by emotion or species observed? (4) Does the viewer's age or sex influence dog gaze? (5) Do dogs and humans visually inspect emotionally expressive individuals (dog and human) in the same way? Although there is no study of human gaze allocation at the full body of dogs, based on previous research focusing on facial expressions, we predicted that human gaze would be affected by both the viewed species and emotional expressions (Guo et al. 2019; Correia-Caeiro et al. 2020) and that both age and sex would modulate gaze patterns at least at human bodies (Pollux et al. 2016). We also predicted that the dog gaze would be affected by the viewed expressions and species since they are able to discriminate and recognise (at least some) prototypical facial expressions (Barber et al. 2016; Correia-Caeiro et al. 2020). No study has looked at perceptual ability variation with age or sex in dogs, but we can speculate that from human research this is probably the case for dogs as well, where age-related cognitive decline and/or experience-based processes can modify face- and body-viewing gaze behaviour. Finally, we predicted that human and dog viewers would both focus on the face primarily as a source of emotional information, since not only faces are important in mammal social communication (Tate et al. 2006), but also all studies published to date focus on how dogs process human faces.

## Methods

### Participants

We recruited 130 humans from the general population (aged 18–86 yo, mean ± SD: 42.7 ± 19.9) and 100 family dogs (aged 2–14 yo, 4.9 ± 2.7) for this study. Nine participants (one human and eight dogs) had their data discarded due to difficulty with tracking their eye movements. Sixty-one participants had between one and five missing trials (due to technical issues with equipment or lack of participant’s attention). Data from 129 humans and 92 dogs were collected successfully (see ESM for more information on recruitment and participants' description).

### Video stimuli

Twenty videos (total duration of all videos: 132.30 s, mean duration of individual clips: 6.30 s, range: 4.87–7.53 s) of humans and dogs displaying spontaneous and naturalistic responses to five emotionally-competent stimuli (relating to the expression of fear, happiness, positive anticipation, frustration and neutral) were played to participants. The four categories of emotion featured in the video stimuli were defined based mainly on the basic emotional mammalian brain circuits sensu (Panksepp 2011, see also Caeiro et al. 2017b) for full description of emotion categories and examples). As control stimuli, we selected videos of neutral/relaxed individuals, i.e. where any specific emotionally triggering stimuli or overt behavioural reactions were absent. These videos were selected from online databases (www.youtube.com and AM-FED database; McDuff et al. 2013) and were chosen on the basis of stimulus quality (e.g. source) and its clear association with an evoked response. Only videos with minimal editing, high image quality (at least 720p), good lighting and visibility of full bodies were selected. The putative emotion eliciting stimulus had to be apparent and clearly identifiable for at least part of the video. By including homemade/amateur videos with naturalistic and spontaneous behaviour we ensured that the responses were ecologically valid, less constricted, and more robust, especially when compared to laboratory studies on emotions. Furthermore, each video was selected to contain the core facial Action Units (AUs) of each emotion that were identified previously in Caeiro et al. (2017b), using the Human FACS (Facial Action Coding System, Ekman et al. 2002) and the DogFACS (Waller et al. 2013) by a trained coder (CC) in both systems. FACS has been the gold standard in human facial behaviour research for over 40 years (Ekman and Friesen 1978) and more recently, also in animal facial behaviour research (Parr et al. 2007, 2010; Caeiro et al. 2013, 2017a; Waller et al. 2015). The anatomically based systematic and standardised tools code independent facial movements in an objective way across different species (Waller et al. 2020), by attributing numerical codes linked to muscular action (AUs, ADs—Action Descriptors and EADs—Ear Action Descriptors) to each movement (e.g. AU101—Inner brow raiser, Waller et al. 2013).

All video stimuli were edited in Adobe Premiere Pro CS6 v6.0.1 to display the full body standardised by the figure height and to fit within vertical calibration points (36°), and to apply a grey (#505050) circular/oval mask to hide most of the background. Videos were also image corrected (gamma, colour balance (HLS) and/or auto colour) whenever needed.

Ten videos featured humans and ten videos featured dogs, in which two videos per emotion and per species were displayed. The same 20 videos were played to all participants in a randomised order.

For more information on video stimuli design, see ESM. For examples of stimuli, see S1 Movie.

### Experimental setup and testing protocol

This experimental setup and testing protocol is identical to that described in Correia-Caeiro et al. (2020). The experiment took place in a dark room (Fig. S1 in ESM) with the stimuli back-projected by an Optoma EX551 DLP projector on a translucent screen (185 cm × 140 cm, 88.67° × 66.35°). An Eyelink 1000 Plus eye-tracker (SR Research Ltd, 500 Hz sampling frequency, 0.25°–0.5° accuracy and 0.01° root-mean-square resolution) in remote mode, placed 60 cm away from both the screen and the participant, collected the allocation of gaze on the stimuli. The face of dog participants and the screen were recorded with two synchronised CCTV night vision cameras during the experiment (Fig. S1 in ESM). For human viewers, after each trial a question appeared on the screen asking participants to freely verbally label the emotion observed after each video, which was then recorded by the experimenter as the Emotion Categorisation Accuracy (ECA). For dogs, free-viewing spontaneous gaze behaviour was recorded. The experimental protocol was slightly different between human and dog participants, to account for species-specific differences, but all participants were displayed the same stimuli.

After signing consent forms, human participants were sat in a chair in front of the screen, placed the target in their foreheads and the eye-tracker was set up. The distance between participant, camera and screen were the same for dog and human participants (Fig. S1 in ESM). The eye-tracker was slightly off-centre for dogs, to be able to track the dog’s eye without the nose blocking the camera view or the IR light. The screen was placed between the participant and the experimenter controlling the eye-tracker to avoid any unconscious cues from the experimenter. The dog participants were lured with a treat or toy to the mat behind the window frame and allowed to spontaneously choose to sit, stand or lie down in front of the owner (or an assistant, if the owner chose not to attend the session). The owner/assistant did not restrain or position the dog in any particular way (i.e. did not physically manipulate nor mechanically force the dog, sensu Alexander et al. 2011). No chin/head rest was used, so the dog's head could freely move behind and within the window frame. The dog was free to choose how to position itself behind the window frame (determined during calibration) and it was free to leave at any point. If the dog chose to leave, the display of the next stimulus would be paused, the experimenter would wait 1–2 min (e.g. for the dog to drink water, walk around the room, etc.) and then call the dog or lure the dog with a treat to come behind the window frame again.

The temporal interval between video display (inter-trial interval) was variable due mainly to the manual drift point correction procedure used (drift points have several important functions, including correcting for large head movements between videos, avoiding central biases, standardising the first fixation on the screen and refocusing the dog's attention). This variation not only accounts for individual variation, but also inter-species variation, since dogs do not promptly fixate dots on a screen, without training. In our study, the drift point was manually recorded because the dogs were not trained specifically to look at the screen, hence the interval would last as long as the dog took to focus an eye for at least 1 s on the target. After each video, the dogs were given a treat, regardless of their behavioural responses or viewing behaviour of the video, i.e. regardless of whether they watched the video or any particular area of the screen. The experimenter giving the treat to the dog could not see the behaviour of the dog during stimulus display. Thus there was no pre-determined behavioural reinforcement during stimulus presentation (i.e. the experimenter could not intentionally reinforce specific behaviours such as looking at a screen or staying still), and more importantly, there was no pre-experiment training to maintain attention to stimulus or stay still. While it is still possible that particular dogs could form an unexpected association between a treat and a particular response in one trial, the randomisation of the video stimuli and our very large and varied sample (different ages, breeds, life history, training skills, etc.) prevents this having a meaningful effect on our data. The treat was used mainly to keep the dog interested and to focus their attention on the drift points. If the dog did not watch more than 50% of the videos (confirmed by looking at the eye-tracking data and video recording of the individual, after finishing the display), the dogs would be played all the stimuli again after a break (~ 15 min). Hence, six dogs were displayed the videos again, with three dogs having no missing trials, and three dogs with 1, 2 and 4 missing trials each. However, the videos already watched on the first display were discarded from the second display during analysis.

### Variables of interest

Three Areas of Interest (AOIs) were defined for the video stimuli using Data Viewer 2.1.1, to investigate participants' gaze behaviour: AOI head, AOI body and AOI window (see further AOI definition and Fig. S2 for AOI examples in ESM). The main variable of interest was the viewing time, which was defined as the summation of the duration across all fixations on each AOI. Because the videos used in this study had different durations and the species tested were likely to show different attention spans, the viewing time at each AOI was standardised as the proportion of viewing time (PVT), i.e. the viewing time at head or body was divided by the total duration of fixations in the AOI window. The AOI window included all the visible stimulus.

As the videos used in this experiment varied in emotional content and the species displayed, the variables of stimulus emotion and stimulus species were used as predictor variables to investigate how they would affect the main response variable (PVT) as well as other participant variables. Additionally, dogs' breed, body size index (calculated by multiplying both body measurements), and cephalic index (brachycephalic, mesaticephalic and dolichocephalic; Ellis et al. 2009) were considered as predictor variables and analysed against the response variables to control for bias in the sample. For both species, age and sex were also used as predictor variables (see ESM for further description of control variables). Finally, for humans, emotion categorisation accuracy (ECA) was recorded and coded as proportions, per participant, per observed species and per observed emotion. For further description of the free-labelling approach, and correct/incorrect labels, see Table S1 and S2 in ESM.

Part of the footage collected from the dogs while they were viewing the stimuli (~ 10%) was fully coded with DogFACS (Waller et al. 2013) by three certified coders in DogFACS (CC and two students not involved in this study: DR and LH) via the open source software BORIS (Behavioural Observation Research Interactive Software) V7.98 (Friard and Gamba 2016). To become certified in DogFACS, coders need to be trained and do a certification test achieving a score of 70% or above, to ensure the coding is standardised and reliable between coders (Waller et al. 2013, www.animalFACS.com). All three coders (CC, DR and LH) successfully passed the DogFACS test, and during coding, were blinded to the category of stimulus being displayed. The number of AUs displayed when viewing each video clip was normalised by the video duration, and the proportion of each AU was used as a response variable to investigate the facial responses to the video stimuli.

### Statistical analysis

Statistical analyses were performed with R 3.4.2. (R Core Team 2017). To understand the distribution of human and dog gaze patterns, GLMMs with binomial family were run for each viewer species with PVT as a response variable, AOI, emotional expression and species as predictors, and participant number nested in case number as a random variable, using glmer function (lme4 R-package). For humans, another binomial GLMM was run, but with age and sex as predictors. To explore the relationship between ECA and the stimuli and the participant variables, further non-parametric tests were run (GLMMs were first run, but did not converge). The relationship between PVT and ECA was also assessed with Kendall’s tau. For dogs, to investigate the effect of age, sex, breed, cephalic index, and body size (see S3 in ESM for body size index description) on their PVT, GLMMs with binomial family were run, with nested random factors of case number in participant number, stimulus species in stimulus emotional expression and stimulus emotional expression in AOI, using the optimiser bobyqa. Finally, to compare the PVT between human and dog viewers, binomial GLMMs were built, with PVT as a response variable, AOI, stimulus emotional expression, stimulus species and participant species as predictor variables, and participant number nested in case number as a random variable. Mann–Whitney tests were used to further explore the effects of the predictor variables in PVT. To investigate the dog facial responses to the emotional cues, a binomial GLMM was run with the proportion of each AU as a response variable, and stimulus emotional expression and stimulus species as predictor variables. All models were compared using AIC (Akaike’s Information Criterion) and ANOVAs. Bonferroni corrections were applied for multiple testing. The uncorrected α value was set at 0.05 for all analysis (see ESM for further information on statistical analysis).

## Results

### Human viewers

**Fig. 1:**
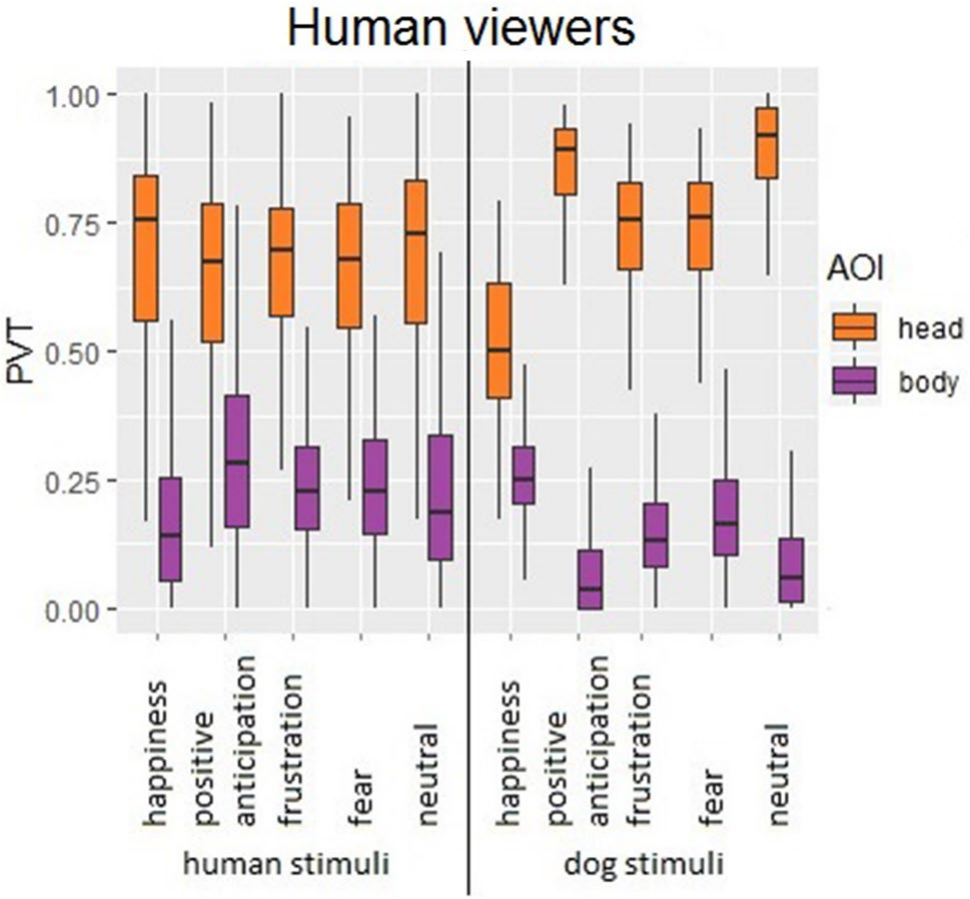
Boxplot with distribution of proportion of viewing time (PVT) in human viewers, by area of interest (AOI), viewed expression, and viewed species. Whiskers represent minimum and maximum, box includes median and interquartile range

Humans displayed the same AOI-dependent viewing pattern to inspect human and dog emotional expressions (Fig. 1 and Table 1), by directing significantly higher PVT at the head than the body of both species (GLMM, *χ*^2^ = − 4.48, *p* = 0.0001; 64.5% ± 23.5 (mean ± SD) vs. 25.4% ± 20.0 for human expressions, 74.0% ± 20.3 vs. 15.2% ± 13.7 for dog expressions). The GLMM showed no species effect, indicating human viewers demonstrated the same gaze distribution in viewing a comparable expression from humans and dogs. Only the expression of happiness modified humans’ viewing pattern (lower PVT than neutral: GLMM, *χ*^2^ = − 1.22, *p* = 0.0001), and for all expressions PVT of the head was higher than for the body (see ESM for posthoc tests and modelling description for human viewers).

**Table 1:** Optimal GLMM model for human viewers’ PVT as a response variable and the predictor variables body region (AOI—head or body) and expression viewed

Predictor variables	Estimate	SE	*z*	*p*
Response variable: PVT				
Intercept	2.0515	0.1133	18.11	0.0001
AOI (body)	− 4.4776	0.1020	− 43.89	0.0001
Expression (happiness)	− 1.2220	0.1452	− 8.42	0.0001
Expression (positive anticipation)	− 0.0774	0.1479	− 0.52	0.6007
Expression (fear)	− 0.2718	0.1472	− 1.85	0.0647
Expression (frustration)	− 0.2595	0.1471	− 1.76	0.0776

**Fig. 2:**
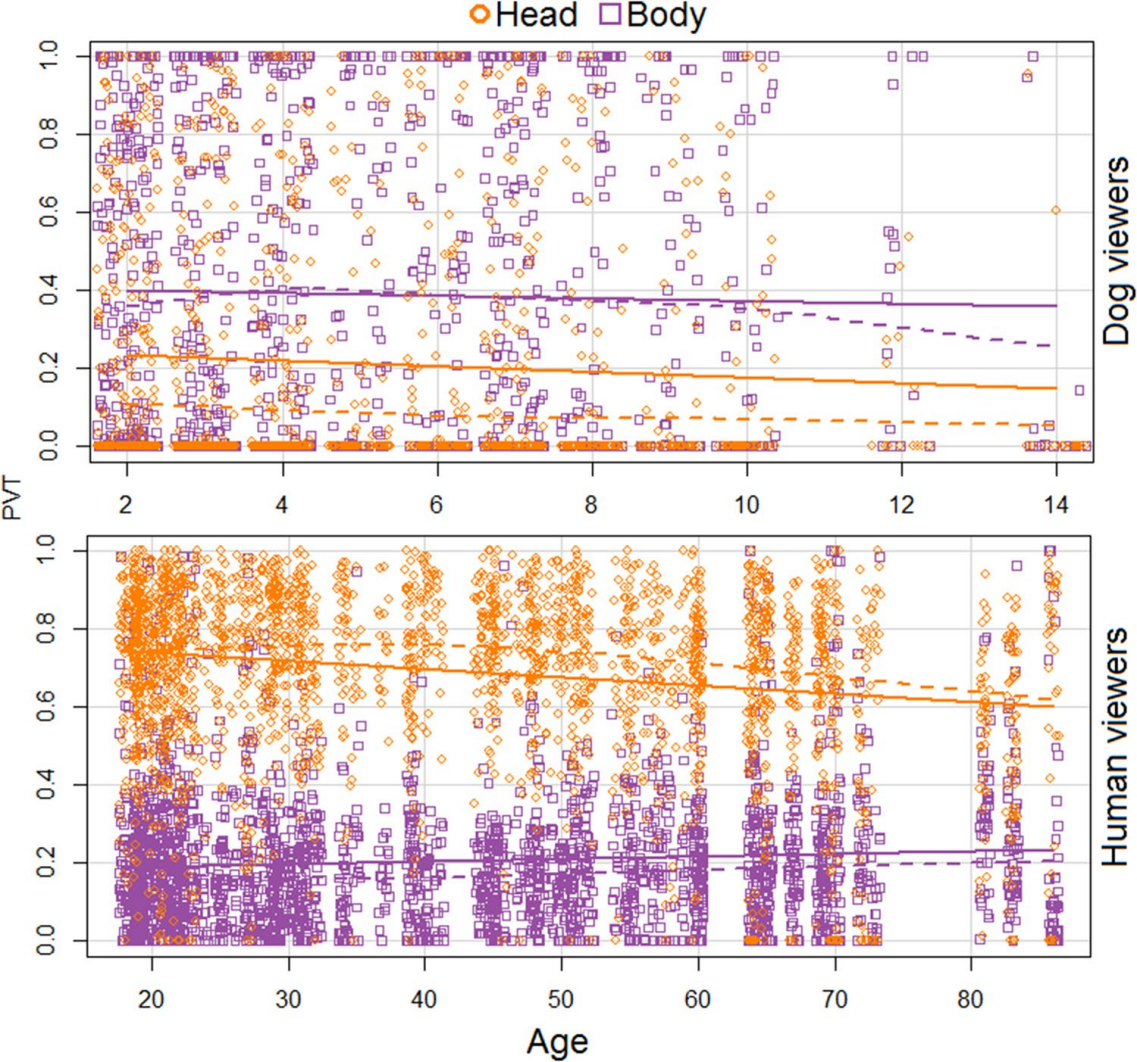
Scatterplot with distribution of the proportion of viewing time (PVT) on the head and body (Areas of Interest—AOI) by human viewers (bottom) and dog viewers (top) across ages. Dashed lines represent mean and solid lines represent variance

Moreover, this viewing pattern was affected by age (GLMM, *χ*^2^ = − 0.008, *p* = 0.0001) but not gender (*χ*^2^ = − 0.017, *p* = 0.83; Table 2). Age negatively correlated with PVT for both human (Kendall’s Tau with Bonferroni correction, *rτ* = − 0.085, *p* = 0.0001) and dog head (*rτ* = − 0.115, *p* = 0.0001), but increased PVT of dog body only (*rτ* = 0.068, *p* = 0.0004; Fig. 2, Fig. S4 in ESM). These age-induced changes in PVT distribution were also correlated within some viewed expressions. Older humans looked less at human heads in positive human expressions (happiness: *rτ* = − 0.14, *p* = 0.0006; positive anticipation: *rτ* = − 0.11, *p* = 0.009), and at dog heads in positive anticipation (*rτ* = − 0.18, *p* = 0.0001), frustration (*rτ* = − 0.12, *p* = 0.004) and neutral (*rτ* = − 0.17, *p* = 0.0001). For dog neutral expressions, older humans tended to look more at the body (*rτ* = 0.16, *p* = 0.0002).

**Table 2:** Optimal GLMM model for PVT as a response variable and the predictor variables age and gender for human viewers

Predictor variables	Estimate	SE	*z*	*p*
Response variable: PVT				
Intercept	− 0.004065	1.206349	− 0.003	0.997
Age (years)	− 0.008477	0.002077	− 4.082	0.0001

Although there was an overall similarity in viewing pattern for human and dog stimuli, human viewers showed significantly higher emotion categorisation accuracy (ECA) for human expressions than for dog expressions (53% ± 43 vs. 37% ± 40; Mann–Whitney, *U* = 2,503,200, *p* = 0.0001). This trend was consistent for all emotion categories except for happiness (Fig. S5 and Table S3 in ESM for ECA distribution and comparison between viewed emotional expression and species). For human expressions, viewers demonstrated the highest ECA for happiness (95% ± 15), followed by fear (84% ± 27), neutral (58% ± 36), frustration (21% ± 29), and then positive anticipation (9% ± 22); whereas for dog expressions, viewers demonstrated the highest ECA for happiness (93% ± 20), followed by neutral (42% ± 33), fear (41% ± 38), positive anticipation (22% ± 29), and finally frustration (2% ± 11). Viewers’ age and gender had little impact on their ECA (Table S4 and S5 in ESM, respectively).

Interestingly, there was a species-modulated correlation between human's viewing pattern and ECA. When recognising human expressions, higher ECA was associated with lower PVT towards body AOI (Kendall’s Tau, with Bonferroni correction, *rτ* = − 0.12, *p* = 0.0001; no association for head: *rτ* = − 0.02, *p* = 0.30); whereas when recognising dog expressions, higher ECA was associated with lower PVT at head AOI (*rτ* = − 0.20, *p* = 0.0001), but higher PVT at body AOI (*rτ* = 0.20, *p* = 0.0001). Thus although humans look proportionally more at the head of both human and dog figures (Fig. 1), their expression recognition performance appears to be related to longer inspection of dog bodily cues, but shorter inspection of human bodily cues.

### Dog viewers

**Fig. 3:**
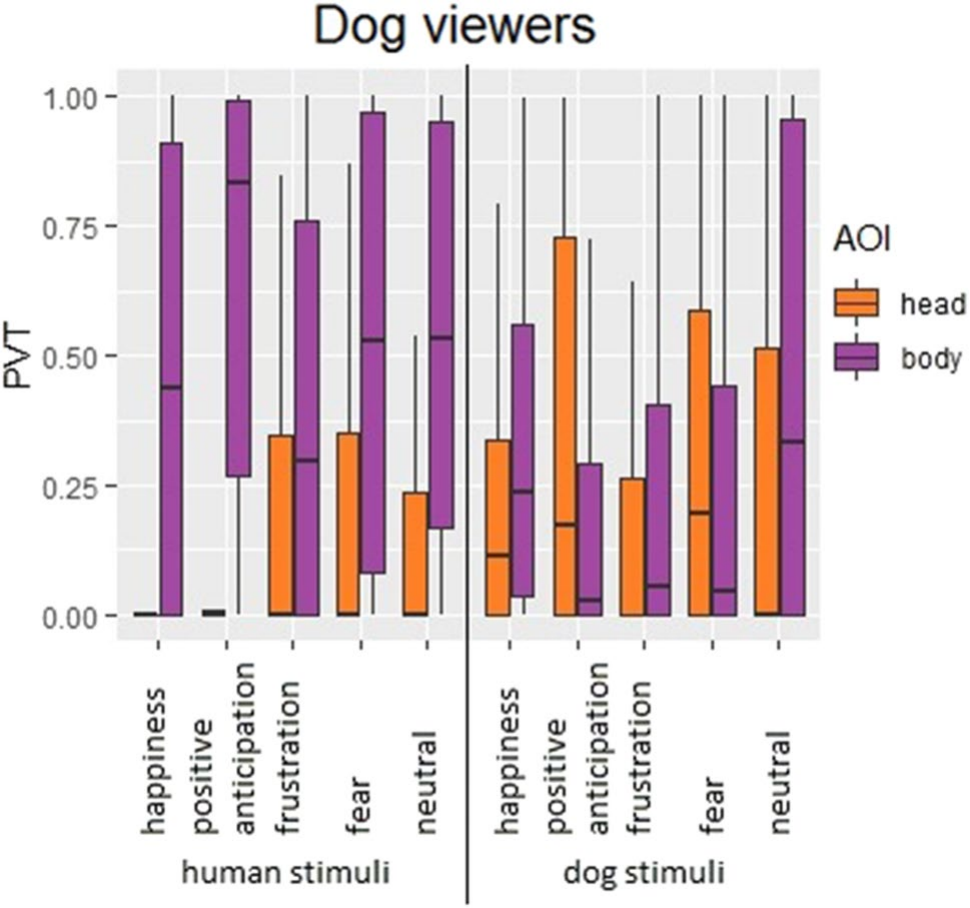
Boxplot with distribution of proportion of viewing time (PVT) in dog viewers, by Area of Interest (AOI), viewed expression, and viewed species. Whiskers represent minimum and maximum, box includes median and interquartile range

Dogs displayed different AOI-, species- and expression-dependent viewing patterns to inspect human and dog emotional expressions (in this order, Fig. 3 and Table 3), by allocating significantly higher PVT at the human body than the human head (52% ± 41 vs. 17% ± 31; Mann–Whitney, *U* = 79,889,000, *p* = 0.0001) and slightly higher PVT at the dog body than the dog head (29% ± 35 vs. 26% ± 34; *U* = 286,793.5, *p* = 0.005). The presented expressions of both species further modified dogs’ viewing pattern to a lesser degree. Specifically, in comparison with the dog head, the dog body attracted higher PVT in happiness (32% ± 30 vs. 22% ± 27, *U* = 10,078.5, *p* = 0.001), frustration (23% ± 31 vs. 18% ± 29, *U* = 10,018.5, *p* = 0.011) and neutral (45% ± 42 vs. 25% ± 36, *U* = 8106.5, *p* = 0.0001), similar PVT in fear (24% ± 33 vs. 32% ± 35, *U* = 14,681.5, *p* = 0.09), and lower PVT in positive anticipation (21% ± 33 vs. 35% ± 37, *U* = 14,271.5, *p* = 0.039); whereas the human body attracted consistently higher PVT than the human head across all expression categories (happiness: 48% ± 41 vs. 22% ± 27, *U* = 3221.5, *p* = 0.0001; positive anticipation: 65% ± 38 vs. 18% ± 29, *U* = 2382, *p* = 0.0001; frustration: 40% ± 39 vs. 18% ± 29, *U* = 3886, *p* = 0.0001; fear: 53% ± 41 vs. 18% ± 29, *U* = 3699, *p* = 0.0001; neutral: 53% ± 40 vs. 25% ± 36, *U* = 2781.5, *p* = 0.0001; see also ESM for modelling description for dog viewers).

**Table 3:** Optimal GLMM model for dog viewer's PVT as a response variable and the predictor variables body region (AOI—head or body), expression and species viewed

Predictor factors	Estimate	SE	*z*	*p*
Response factor: PVT				
Intercept	− 0.47094	0.10750	− 4.381	0.0001
AOI (head)	− 0.76550	0.08634	− 8.866	0.0001
Expression (happiness)	− 0.40783	0.13531	− 3.014	0.0026
Expression (positive anticipation)	0.05008	0.12957	0.387	0.6991
Expression (fear)	− 0.23673	0.13161	− 1.799	0.0721
Expression (frustration)	− 0.49439	0.13987	− 3.535	0.0004
Species (human)	0.43648	0.08560	5.099	0.0001

Similar to human viewers, dogs’ viewing pattern was affected by age (χ^2^ = − 0.04, *p* = 0.008, Fig. 2 and Table 4) but not sex, cephalic index, body size (Table S6 in ESM) nor breed (see ESM for modelling description and breed posthoc analysis). Increasing age resulted in decreased PVT of the head (*rτ* = − 0.015, *p* = 0.016) irrespective of the viewed expression and species (Fig. 2; Fig. S6 in ESM).

**Table 4:** Optimal GLMM model for dog viewers’ PVT as a response variable and the predictor variable age

Predictor factors	Estimate	SE	*z*	*p*
Response factor: PVT				
Intercept	− 0.66597	0.31590	− 2.108	0.0350
Age (years)	− 0.04473	0.01683	− 2.658	0.0079

Humans often show spontaneous facial mimicry when viewing other human expressions as part of both processing and communicating facial movement (Sato and Yoshikawa 2007). To examine whether dogs might similarly process the viewed emotional content (i.e. as an analogue of the analysis of human ECA), we analysed whether their own facial responses were associated with the viewed human and dog emotional expressions using DogFACS (Waller et al. 2013). No differences in facial responses were found between viewed emotion categories. However, dog viewers tended to turn their heads left more often when viewing human rather than dog emotions (Action Descriptor 51 (AD51)—Head turn left, GLMM: χ^2^ = − 0.009, *p* = 0.046, Table 5) and brought their ears closer together more often when viewing dog rather than human emotions (Ear Action Descriptor 102 (EAD102)—Ears adductor, χ^2^ = 0.009, *p* = 0.018, Table 6).

**Table 5:** Optimal GLMM model for dog viewers’ AD51—Head turn left as a response variable and the predictor variable viewed species

Predictor factors	Estimate	SE	*z*	*p*
Response factor: AD51 Head turn left				
Intercept	− 0.043	0.015	− 2.893	0.004
Species (dog)	− 0.009	0.004	− 2.003	0.046

**Table 6:** Optimal GLMM model for dog viewers’ EAD102—Ears adductor as a response variable and the predictor variable viewed species

Predictor factors	Estimate	SE	*z*	*p*
Response factor: EAD102 Ears adductor				
Intercept	− 0.055	0.013	− 4.386	0.0001
Species (dog)	0.009	0.004	2.369	0.0180

### Comparison of human and dog viewers

**Fig. 4:**
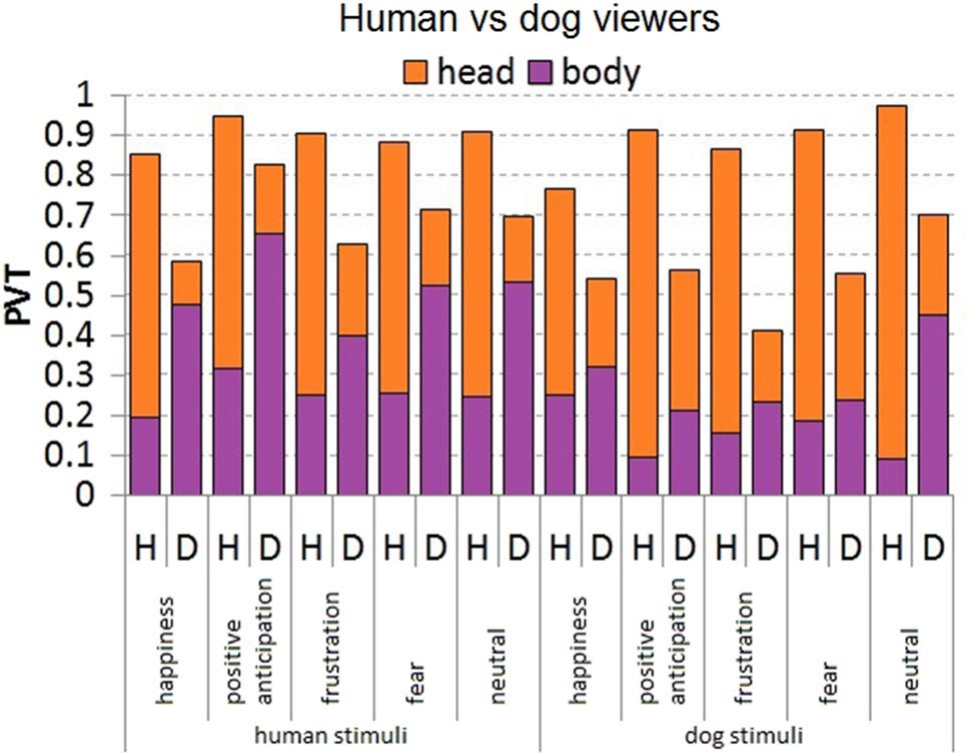
Distribution of proportion of viewing time (PVT) in human (H) vs. dog (D) viewers, by Area of Interest (AOI, head and body), emotion and stimulus species

To address whether dogs and humans use similar viewing patterns to process emotional expressions, we modelled PVT distribution for viewer species, and the viewed AOI, species and emotion categories. The PVT patterns were strongly affected by the viewed AOI (GLMM: χ^2^ = 2.15, *p* = 0.0001, Table 7) and the viewer species (χ^2^ = 0.79, *p* = 0.0001), but less so by the viewed species (χ^2^ = 0.22, *p* = 0.0001) and emotion categories (happiness: χ^2^ = − 0.61, *p* = 0.0001; positive anticipation: χ^2^ = − 0.002, *p* = 0.98; frustration: χ^2^ = − 0.26, *p* = 0.0016; fear: χ^2^ = − 0.18, *p* = 0.03). The direct comparison of viewers’ PVT distribution for each viewed expression and species (Fig. 4) revealed that in comparison to human viewers, dog viewers tended to show a shorter overall PVT (31% ± 37 vs. 45% ± 32; χ^2^ = 0.79, *p* = 0.0001) and different PVT distributions with shorter PVT of the head (22% ± 33 vs. 70% ± 22; *U* = 544,070, *p* = 0.0001) but longer PVT of the body AOI (39% ± 39 vs. 20% ± 18; *U* = 1,936,200, *p* = 0.0001). Such differences in viewing pattern between dogs and humans were more evident when inspecting human expressions. Specifically, dogs looked more at the human body than human viewers (52% ± 40 vs. 25% ± 20; *U* = 487,400, *p* = 0.0001) for all presented expressions except for frustration, whereas humans looked more at the human head than dog viewers (65% ± 23 vs. 17% ± 31; *U* = 108,920, *p* = 0.0001). When inspecting dog expressions, dogs and humans directed indistinguishable PVT at dog bodies (29% ± 35 vs. 15% ± 14; *U* = 501,750, *p* = 0.36), but humans still looked more at the dog head than dog viewers (74% ± 20 vs. 26% ± 34; *U* = 149,230, *p* = 0.0001) for each of the presented expressions (see also ESM for modelling description, and post hoc tests and mean ± SD in Tables S7, S8, respectively; see Movie S1 for a comparison of human and dog viewers gaze). Total looking times also differed greatly between humans and dogs, with humans focusing on the stimuli for much longer than dogs (Mann–Whitney, *U* = 4,470,078.5, *p* = 0.0001, see Table S9 in ESM for mean ± SD per viewed expression and species).

**Table 7:** Optimal GLMM model for human and dog viewer's PVT as a response variable and the predictor variables viewer's species, body region (AOI—head or body), expression and species viewed

Predictor factors	Estimate	SE	*Z*	*p*
Response factor: PVT				
Intercept	− 2.0391	0.083160	− 24.52	0.0001
Viewer's species (human)	0.78993	0.057353	13.77	0.0001
AOI (head)	2.15006	0.055479	38.75	0.0001
Viewed species (human)	0.217760	0.053306	4.09	0.0001
Expression (positive anticipation)	− 0.002287	0.082488	− 0.03	0.9778
Expression (happiness)	− 0.606617	0.085891	− 7.06	0.0001
Expression (frustration)	− 0.263972	0.083562	− 3.16	0.0016
Expression (fear)	− 0.179316	0.082630	− 2.17	0.0300

## Discussion

In this study, we found that humans displayed the same gaze distribution (i.e. longer viewing time at the head compared to body regions) in viewing dynamic human and dog emotional expressions of different categories, indicating humans preferentially focus on facial expressive cues to judge human and dog emotional states even though bodily cues might be more overt. This observation is in broad agreement with previous findings that humans have similar viewing behaviour and similar brain regions activation (Kujala et al. 2012; Desmet et al. 2017), even though there are notable differences in appearance and actions performed between species. Hence, these results suggest that humans do not employ a flexible viewing strategy varying according to species viewed, i.e. humans typically inspect any species as if they were humans, even for emotional expressions (Schirmer et al. 2013; Konok et al. 2015). While this might confer some advantages in the human–dog relationship (e.g. higher empathy (Kujala et al. 2017)), it may not be fully adaptive given differences between human and dog facial cues (Caeiro et al. 2017b), and dogs' conspicuous use of body parts absent from humans (Bradshaw and Nott 1995). This may, in part, explain the poor human performance in correctly identifying dog emotional expressions and behaviours (Horowitz 2009; Demirbas et al. 2016). If humans look at dogs as if they were humans, they probably miss important cues.

Age-related changes in the perception of emotional cues have been widely reported for both facial (Sullivan et al. 2017) and bodily expressions (Pollux et al. 2016). While several studies have shown a general decline in emotion recognition associated with age for most emotions (Kret and Gelder 2012; Sullivan et al. 2017), in our study sex or age of viewers had little impact on ECA. It is still not clear if ageing effects are simply due to cognitive decline, or changes in emotion regulation strategies modulated by factors such as life experience, motivational goals and/or structural brain changes. However, in our study, we found that the strong human attentional preference to the face was affected by ageing, with increased focus on the body. Facial cues in humans are regarded as more determinant of emotion category, while bodily cues of emotion intensity (Ekman and Friesen 1967). Therefore, this age-related change might mean an increased focus on the intensity of categorised emotion. Perhaps surprisingly, higher head PVT was associated with lower ECA, while higher body PVT was related to higher ECA. This could simply reflect differences in competence (i.e. those more able at ECA need less time to process the face, and so spend more time on other regions providing important adjunctive information, such as intensity of emotion).

In contrast to human viewers, dogs allocated longer viewing times to the body than the head region in both human and dog dynamic emotional expressions. Dogs excel at detecting subtle human facial behaviour, such as facial expressions (Nagasawa et al. 2011; Buttelmann and Tomasello 2013; Müller et al. 2015), but little is known about how dogs perceive whole bodies in an emotional context. Our data provide the first empirical evidence that bodies are not only important elements of social cues for dogs, but are more important than heads, when interacting with either conspecifics or humans. Therefore, the main conclusion of our study is that bodily emotional expressions are a primary source of information for dogs, while facial expressions are secondary, which refutes previous assumptions of face-centric interactions being most important between humans and dogs (Gácsi et al. 2004; Jakovcevic et al. 2012). However, this dog gaze pattern contrasts with humans and non-human primates' gaze behaviour (Kano and Tomonaga 2009) which focus primarily on the face during emotional expressions. Furthermore, dogs' gaze allocation was affected by the viewed species and emotions, suggesting greater cue-related gaze behaviour flexibility in dogs than in humans.

Like humans, dogs’ gaze allocation was affected by ageing, with reduced viewing of the head but not the body. Unlike humans, head and body PVT was not correlated in dogs. This age-related effect may be linked to a difficulty in maintaining attention with age (Chapagain et al. 2017), alongside other possible factors, such as cumulative experience effects (older dogs have more experience so need less time to read cues). Dogs displayed some differential FACS coded facial movements when observing human and dog emotional expressions, but no evidence of facial mimicry. The lateralised bias response in dogs has been reported before in relation to both emotional faces (left gaze bias, Racca et al. 2012) and specific threatening stimuli (left head-turning, Siniscalchi et al. 2010). It has been suggested this is because facial and/or emotional stimuli may be preferentially processed by the right cerebral hemisphere. However, our results indicate a bias in this behaviour when viewing human expressions and the reasons for this are not entirely clear. Dogs also tended to produce more EAD102—Ears adductor when observing dog than human figures, which has been found before to be linked to a positive anticipation context (Caeiro et al. 2017b; Bremhorst et al. 2019). This indicates dogs had a positive response to observe dogs on the screen, and perhaps a more negative response to the unfamiliar humans. Nevertheless, these differential facial actions suggest that dogs do not just passively view the presented emotional stimuli, but show some level of functional response or understanding of (at least) the species observed.

Human and dog viewers not only differed significantly in their overall PVT, but also in their viewing of: (1) head vs. body of individuals, with humans looking longer at the head than dogs, and dogs looking longer at the body than humans (i.e., not only do humans look more at the head than the body, but they also look more at the head than dogs, with the opposite occurring in dog viewers); (2) conspecifics vs. heterospecifics, except when observing dogs’ bodies; more specifically, the head of both human and dog figures attracted higher PVT from human than dog viewers; (3) most emotion categories, with higher head PVT but lower body PVT from human than dog viewers. Hence, while it seems that humans read other species as if they were humans, dogs present more varied perceptual strategies depending on the species observed.

One possible explanation for dogs’ attention towards bodies might be related to low-level saliency of cues (e.g. size) and relative position in social interactions between humans and dogs, i.e., bodies are larger than heads and are closer to the eye level of dogs. Given these differences between humans and dogs, the cross-species perception of emotional cues might be strikingly different, and thus explain the results found in this study, particularly when viewing humans. Even though bodies got most of the PVT, dogs still looked at faces for approximately 23% of the time (vs. 39% for the body), which means faces are still a relevant stimuli for dogs, but bodies seem to be more visually relevant.

Early eye-tracking studies often normalise or standardise eye gaze data according to the defined AOI size to control for the so-called ‘uniform looking strategy’ which argues that gaze duration at a given AOI may be determined by its size (Dahl et al. 2009). However, numerous research studies on both humans and non-human animals have revealed that gaze allocation in viewing of (at least) biologically relevant stimuli (e.g. faces and bodies) is driven by task-relevant or situation-related diagnostic information contained in local image regions rather than AOI sizes or low-level local image saliency, such as local luminance or colour contrast (e.g. Guo et al. 2019). Hence a ‘uniform looking strategy’ is not applicable in social attention research. While standardising AOI size may help to clarify the minor effect of changing AOI sizes (e.g. smiling mouth vs. neutral mouth, human mouth vs. dog mouth) on gaze allocation, it can bias or even misinterpret research findings especially with larger differences between AOI sizes. Taking body perception research as one example, as heads are much smaller than bodies, standardising AOI size would artificially overestimate the role of face/head in body perception and ignore the important role of bodily expression in emotion perception (e.g. Pollux et al. 2019) and body size/attractiveness judgement (e.g. Rodway et al. 2019). Despite further studies being needed to exclude low-level effects completely, high-level aspects are more likely to explain our results, due to dogs' behavioural repertoire: when dogs interact with conspecifics, they do not spend much time face to face, instead placing themselves more laterally to each other (Rooney and Bradshaw 2002), and they usually inspect each other’s body (mostly for odour recognition, Rooney and Bradshaw 2002), but not faces. In dogs, a fixed stare is also part of agonistic displays (McGreevy et al. 2012), and hence dogs might generally avoid prolonged gaze at faces. Therefore, dogs might have adapted their behavioural repertoire, particularly their initial social evaluation strategy of a conspecific for interaction with humans.

Humans and dogs also differed significantly in their total viewing time of the stimuli, with humans observing the stimuli for much longer than dogs. The explanation for this marked species difference is not well understood, and it could be argued that the dogs simply lost interest in the stimuli in the adopted free-viewing task. However, the relatively short viewing time at visual stimuli has been commonly reported in other dog visual perception studies (Guo et al. 2009; Racca et al. 2012; Törnqvist et al. 2015), even when the dog was trained to look at the stimuli for the total duration of the display (Törnqvist et al. 2015). Hence, this explanation seems less plausible, and instead, other explanations such as dogs having shorter attention spans, quicker processing of information, or less information extracted seem more plausible.

In our study, the experimental protocol was designed to obtain naturalistic and unconditioned responses, but also to account for differences between the species. One difference between our protocol for humans and dogs that might be relevant to how eye movements are produced, was the task goal. Human viewers were asked to identify the emotion after viewing the stimuli, while dog viewers were simply free-viewing the stimuli. It is well known in the eye-tracking literature that the nature of the cognitive task results in gaze patterns differences due to top-down factors, i.e. given a particular task, the eyes fixate on more informative locations for the task required, while in free-viewing low-level saliency (e.g. high contrast areas) may attract more fixations (Yarbus 1967; Borji and Itti 2014). However, it is also well established that emotional stimuli engage attention and activate motivational and action systems, while neutral pictures do not (Lang and Bradley 2010; Bradley et al. 2011; Mastria et al. 2017). Since emotional expression is one of the most salient aspects of a social stimulus, asking viewers to explicitly identify the emotion viewed is probably a similar process to the implicit emotion processing in free-viewing. In both human and non-human primates, social relevance but not low-level aspects, drives viewing behaviour (Solyst and Buffalo 2014), but for dogs this is not yet known. Nonetheless, although we do not know for sure if emotion cues are the most salient aspects of a social stimuli for dogs, we know they recognise facial expressions of emotions both in conspecifics and humans (Albuquerque et al. 2016) and that emotion cues for dogs, as in all mammals (Tate et al. 2006) are crucial for fitness and survival. Hence top-down processes are more likely to take priority for both humans and dogs, i.e. in free-viewing it is likely that dogs, like humans, focus on emotion cues, but future studies should empirically test these predictions.

We have reported previously no commonality between humans and dogs in facial responses to emotional triggers (Caeiro et al. 2017b). We observed little commonality in gaze allocation used for extracting diagnostic expressive cues between these two species, both in faces [see previous study, (Correia-Caeiro et al. 2020)] and full bodies (present study). Although there might be an ancient mammalian homology in facial musculature and in the neural system sub-serving emotion processing between humans and dogs (Leopold and Rhodes 2010; Schirmer and Adolphs 2017), our work shows that both expression and perception of emotional cues varies widely in two species that have shared the same ecological niche for thousands of years. These results challenge the universality of emotion expression in mammals postulated by Darwin (Darwin 1896), suggesting instead that homologies are only anatomical, but not behavioural, and thus not mechanistic.

## Supplementary Information

Below is the link to the electronic supplementary material.

Supplementary file1 (DOCX 382 KB)

Supplementary file2 (XLSX 827 KB)

Supplementary file3 (MP4 51066 KB)
