# Supplementary material for: Bodily emotional expressions are a primary source of information for dogs, but not for humans
Source: Anim Cogn. 2021 Jan 28;24(2):267–79. doi: 10.1007/s10071-021-01471-x (PMC8035094; doi:10.1007/s10071-021-01471-x)
Supplement: Supplementary file 1 — Supplementary file1 (DOCX 382 KB) [file 10071_2021_1471_MOESM1_ESM.docx]

**Electronic Supplementary Materials (ESM) for research article:**

Bodily emotional expressions are a primary source of information for dogs, but not for humans

Catia Correia-Caeiro,^1,2,3^* Kun Guo,^1^ Daniel Mills^2^

^1^School of Psychology, University of Lincoln, UK.

^2^School of Life Sciences, University of Lincoln, UK.

^3^Primate Research Institute, Kyoto University, Japan (current address).

***Corresponding author**:[catia_caeiro@hotmail.com](mailto:catia_caeiro@hotmail.com)

**Methods**

**(a) Participants**

**Participants’ recruitment:** Advertisements were posted in the university news page, in local social media groups and local businesses to recruit humans and dogs. A collaboration with the University of the 3rd Age and a University Patient and Public Involvement representative ensured that older adults were represented in the sample. Human participants working as academics were excluded in order not to bias the sample. Dogs were also recruited directly from the University pet-owner database PetsCanDo (www.lincolnpetscando.co.uk). Human participants and dog owners received a £5 voucher after study completion. The only exclusion criterion for humans was known uncorrected visual deficits. Exclusion criteria for dogs were known or overt vision issues or aggressive behaviour towards unfamiliar people.

**Participants’ description:**

- Human participants included 102 females and 27 males. The cultural groups were distributed as follows: 1 white American, 1 Argentinean, 1 Brazilian, 1 mixed British, 1 oriental British, 110 white British, 12 white Europeans, 1 black Irish, 1 middle eastern.

- Dog participants consisted of 43 females and 49 males. The distribution of breeds were as follows: 9 Border Collie, 2 Belgian Malinois, 4 Border Terrier, 2 Bichon Frise, 5 Cocker Spaniel, 1 Chihuahua, 9 Golden Retriever, 1 German Shepherd, 3 German Shorthaired Pointer, 1 Greyhound, 2 Hungarian Vizsla, 2 Jack Russell, 1 Japanese Spitz,1 Kelpie, 11 Labrador Retriever, 2 Large Munsterlander, 5 Miniature Schnauzer, 21 mixed breed, 1 Papillon, 1 Parson Russell Terrier, 1 Poodle, 1 Pug, 1 Smooth Collie, 1 Staffordshire Bull Terrier, 3 Whippet.

**(b) Video stimuli:**

The 20 video stimuli varied in duration (5-7s) due to inherent variation in the display of spontaneous naturalistic emotional expressions. Videos were selected to contain core facial actions for each context (see (Caeiro et al. 2017)), but no particular body actions were selected. Since body actions for emotions are not considered universal (Ekman 1977; Gelder 2009), and can be widely variable (Winters 2005; Kleinsmith et al. 2006), they were not coded in the stimuli. The video stimuli were selected to ensure there was body movement present in at least some of the videos, in order to make it more comparable to the facial movements, but not any particular type of body movement. All videos had different individuals and included adult men, women and dogs extracted from research databases and YouTube (see Caeiro et al. 2017 for more information on criteria for video selection). The experiment was designed and displayed on Experiment Builder v.1.10.1630 (SR Research Ldt).

**
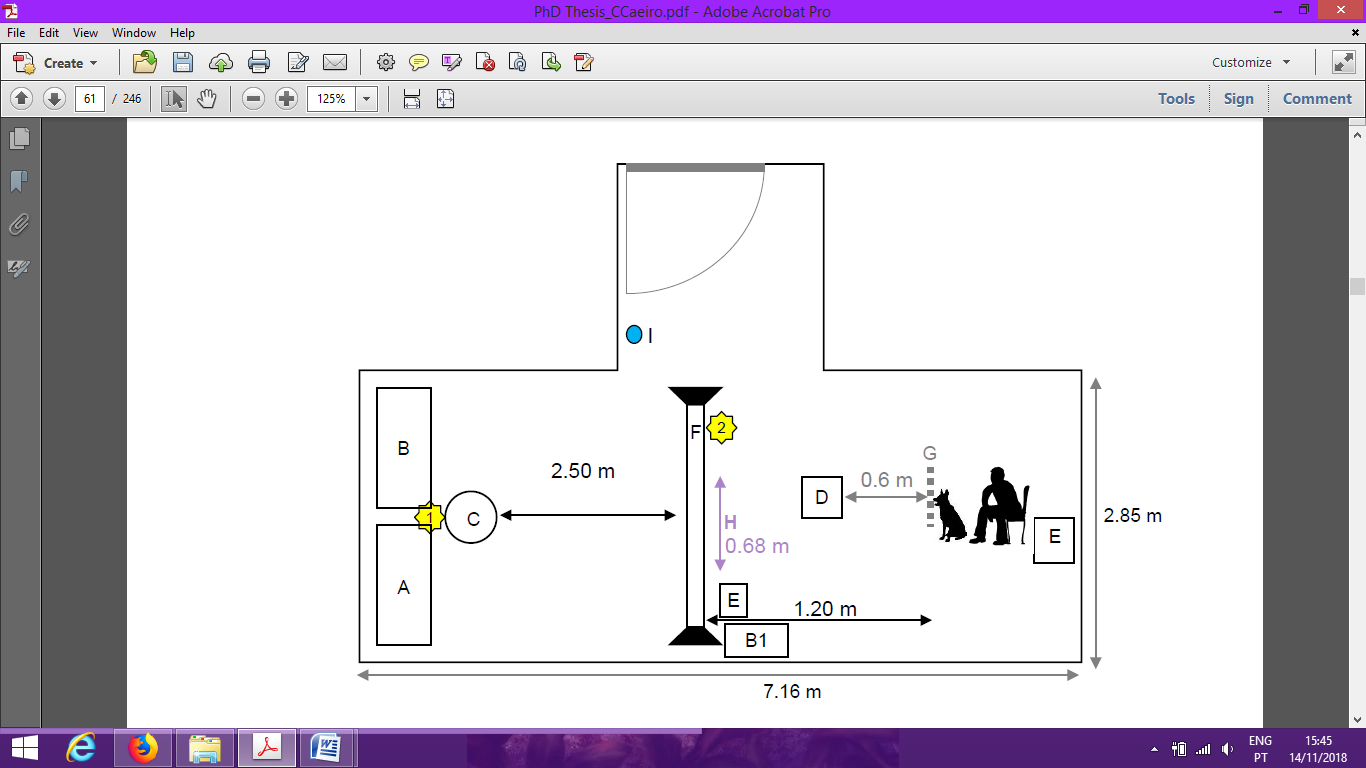
**

**Fig. S1.** Test room diagram. A – Cameras synchronizer and screen; B – Eye-tracker Host Laptop; B1 – 2nd screen of eye-tracker host laptop; C – Projector, display computer; D – Eye-tracker camera; E – Video cameras; F – Screen, G – Window frame, H - Maximum width of stimuli, I - Water bowl, 1 – Experimenter 1 (not visible to participant), 2 – Experimenter 2 (back visible to participant).

**(d) Variables of interest:**

**AOIs:** Although it is common to standardize the eye movement metric by the AOI size (e.g. (Somppi et al. 2014)) this was not performed in this study because the stimuli displayed was close to life-like size, and our primary focus was on responses in an ecologically valid setting. Standardizing per AOI size would disrupt the natural proportion of head/body, making the response variable lose its ecological validity. These AOIs were researcher-defined, hypothesis-driven and followed suggestions for noise-reduction (Hessels et al. 2016).

**
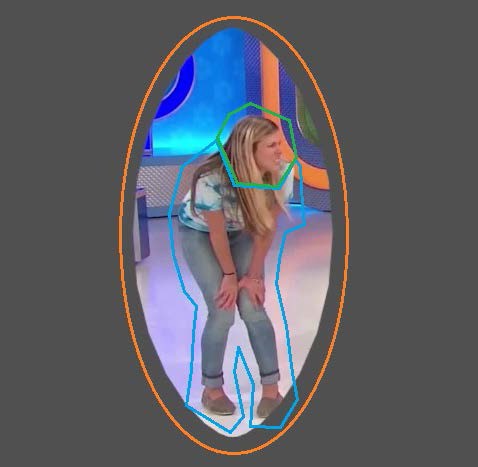

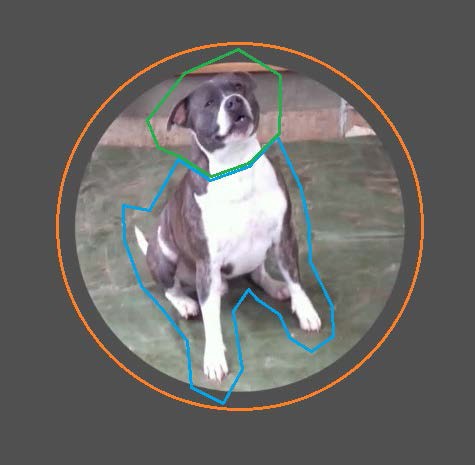
**

**Fig. S2.** Example of body regions (AOIs) in a human participant and a dog participant. In orange is the AOI window, in green is the AOI head and in blue is the AOI body.

**Control variables on human and dog background:** a range of variables were collected from all participants and analyzed against PVT and ECA in order to control for bias in our sample. These variables were selected due to previous literature suggesting associations with either conspecific or heterospecific communication and emotional expression/perception. Namely, for dogs, we collected breed, body size index, cephalic index, and selected scores for the Dog Personality Questionnaire (DPQ; Jones 2008) and the Positive and Negative Activation Scale for dogs (PANAS, Sheppard and Mills 2002). Likewise, for humans, several factors known to influence emotion perception were extracted from a questionnaire administered to participants after the experiment, which included amount of sleep (Tempesta et al. 2010), participant’s cultural group (Matsumoto 1993), medical conditions (including variables related to mental health; Kohler et al. 2011; Phillips et al. 2003) and likeability/experience with dogs (Kujala 2017; Wan et al. 2012). All analysis between PVT and these variables were non-significant after Bonferroni correction. We found that ECA for dog facial expressions of happiness differed significantly between human participants with (0.76 ± 0.31) and without face avoidance (0.95 ± 0.17), i.e. difficulty in looking at other peoples’ faces (Mann-Whitney with Bonferroni correction, U=940.5, p=.0001).

**ECA (Emotion Categorization Accuracy):** In this study, a free-labelling approach was chosen to categorize the emotions observed in order to create a more naturalistic evaluation of the emotions observed and to avoid incurring in an artificially increased recognition accuracy from a forced-choice paradigm, which is the more common approach in studies of emotion perception (Frank and Stennett 2001). Free-labelling was also preferable for cross-species categorization of emotion, to reduce anthropomorphic categorization of emotions. Participants' labels for each emotion varied widely, both for human and dog stimuli, featuring several idiosyncratic responses. The labels considered correct and incorrect are presented in Tables S1 and S2 respectively. As the emotion categorization with free labelling is known to produce a variety of responses even in the same species and widely validated stimuli (e.g. Russel et al. 1993), and in this study inter-species emotion categorization was analyzed, the approach used by Izard (1971) was here applied. Thus, loosely related words to each emotion were also considered correct, as long as the meaning was still linked to that emotion. Previous work by Plutchik (1980) and more recent work by Ekman & Ekman (2017) was used to classify the words within emotion categories.

**Table S1.** Words or expressions (labels) from human participants that correctly categorized the five emotions of the observed stimuli with the corresponding number of times it was used for that emotion between parentheses. For each emotion it is also given in parentheses the total number of trials for all participants, excluding blank answers.

| **Happiness (460)** | **Positive anticipation (510)** | | **Fear (511)** | **Frustration (512)** | | | **Relaxed (512)** |
| --- | --- | --- | --- | --- | --- | --- | --- |
| excitement (141) | positive anticipation (28) | | nervousness (78) | disappointment (26) | | | neutral (99) |
| happiness (132) | waiting to be given something (15) | | scare (75) | frustration (15) | | | relaxed (26) |
| playfulness (78) | curiosity (12) | | anxiety (42) | impatience (9) | | | no emotion (25) |
| joy (17) | interest (7) | | fright (36) | wanting someone to do something but not happening (1) | | | calm (17) |
| pleased (11) | expectation (4) | | shock (28) | getting message across but couldn’t do it (1) | | | alert (10) |
| fun (6) | inquisition (3) | | fear (19) | sad like expecting something but didn’t turn up/ negative (1) | | | talking or communicating (7) |
| elation (6) | looking for something (3) | | startle (8) |  | | | thinking (7) |
| ecstatic (6) | eager to... (2) | | worry (5) |  | | | listening (5) |
| energy (5) | hope (2) | | apprehension (3) |  | | | patience (4) |
| content (4) | wonder (1) | | agitation (2) |  | | | concentration (4) |
| delight (4) | preparing (1) | | concern (2) |  | | | asking for  attention (3) |
| enthusiasm (4) | looking for affection (1) | | cautious (2) |  | | | contemplation (2) |
| enjoyment (3) | nosiness (1) | | alarm (2) |  | | | indifference (2) |
| exuberance (3) | excited with anticipation (1) | | wary (2) |  | | | pensive (2) |
| triumph (2) | waiting for next move (1) | | expected to be told off (negative anticipation) (2) |  | | | watchful (2) |
| pride (2) | waiting forward (1) | | distress (2) |  | | | instructing (2) |
| manic (1) | intrigue (1) | | unease (2) |  | | | chilled (2) |
| amusement (1) | willing (1) | | on edge (1) |  | | | peaceful (2) |
| mischievous (1) | anticipation food (1) | | cowering (1) |  | | | looking around (2) |
| feeling good (1) | trying to get something/ good situation (1) | | cringe (1) |  | | | wondering  about (2) |
| carefree (1) |  | | looking over  with care (1) | | | | disinterested (2) |
| boast (1) |  | | intimidation (1) | | | | making a decision about what to do next (1) |
| humorous (1) |  | | avoidant (1) | | |  | nothing (1) |
| jubilation (1) |  | | panic (1) | | |  | wanting  something (1) |
| smile (1) |  | |  | |  | | "look at me" (1) |
| bouncy (1) |  | |  | | |  | steady state (1) |
| thrilled (1) |  | |  | | |  | apathetic (1) |
|  |  | |  | | | | describing (1) |
|  | |  |  | | | | fixation (1) |
|  | |  |  | | |  | understanding (1) |
|  | | |  | | |  | comfort (1) |
|  | |  |  | | |  | at ease (1) |
|  | |  |  | | |  | messing around (1) |
|  | |  |  | | |  | pointing (1) |
|  | |  |  | | |  | bending (1) |
|  | |  |  | | |  | what is happening on the side of me (1) |
|  | |  |  | | |  | reaching (1) |
|  | |  |  | | |  | unimpressed (1) |
|  | |  |  | | |  | unbothered (1) |
|  | |  |  | | |  | ignorant (1) |
|  | |  |  | | |  | nonchalant (1) |
|  | |  |  | | |  | discussing (1) |
|  | |  |  | | |  | carefree (1) |
|  | |  |  | | |  | normal (1) |
|  | |  |  | | |  | passivity (1) |
|  | |  |  | | |  | dormancy (1) |

**Table S2.** Words or expressions from human participants that incorrectly categorized the five emotions of the observed stimuli with the corresponding number of times it was used for that emotion between parentheses.

| **Happiness** | **Positive anticipation** | **Fear** | **Frustration** | **Relaxed** |
| --- | --- | --- | --- | --- |
| peace (5) | excitement (79) | surprise (36) | happy (60) | sad/unhappy (29) |
| anxiety (2) | nervousness (42) | tired (30) | neutral (48) | sleepy (28) |
| positive anticipation (2) | neutral (35) | sad/unhappy (30) | no emotion (36) | tired (28) |
| curious (2) | happiness (32) | neutral (12) | unhappy/sad (29) | unsure (22) |
| frustrated (2) | anxious (18) | stress (9) | annoyed (28) | bored (19) |
| waiting (2) | hungry/thirsty (12) | guilty (8) | embarrassed (27) | content (17) |
| inquisitive (1) | no emotion (12) | unsure (5) | playful (14) | nervous (15) |
| irritated (1) | unsure (10) | hot (5) | relaxed (14) | attentive (10) |
| nervous (1) | impatient (9) | exhausted (4) | unsure (12) | wanting (9) |
| sad (1) | attentive (9) | feeling sorry (4) | curious (11) | confident (6) |
| tired (1) | apprehensive (9) | submission (3) | tired (11) | anxious (5) |
| trying to bite (1) | sad/unhappy (9) | determined (3) | anxiety (9) | looking for attention (4) |
| unsure (1) | discomfort (7) | discomfort (3) | something (8) | confused (4) |
| no emotion (1) | listening (7) | timid (3) | in pain (7) | hesitant (3) |
| anticipation (1) | worried (7) | relaxed (2) | disgust (6) | anticipation (3) |
| confused (1) | agitated (7) | resigned (2) | excitement (6) | questioning (3) |
|  | calm (6) | boredom (2) | interest(6) | worried (3) |
|  | confused (6) | calm (2) | confused (5) | depressed (2) |
|  | playful (5) | content (2) | anticipation (5) | lazy (2) |
|  | bored (5) | ashamed (2) | calm (5) | patient (2) |
|  | concerned (5) | hope (1) | waiting (5) | submissive (2) |
|  | patient (5) | pleasure (1) | boredom (4) | weary (2) |
|  | pleased (5) | humiliated (1) | worried (4) | cautious (2) |
|  | relaxed (5) | humble (1) | submissive (3) | fear (2) |
|  | tense (5) | hungry/thirsty (1) | upset (3) | angry (2) |
|  | thinking (5) | in pain (1) | wanting something (3) | apprehensive (2) |
|  | guilty (4) | lovely (1) | wary (3) | wanting to be hugged (1) |
|  | alert (4) | no emotion (1) | lonely (3) | annoyed (1) |
|  | submissive (4) | nothing (1) | fed up (3) | aggressive (1) |
|  | stress (3) | obedient (1) | friendly (3) | busy (1) |
|  | focused (3) | attentive (1) | communicating (3) | conflicted (1) |
|  | friendly (3) | aware (1) | confident (3) | contempt (1) |
|  | intensive (3) | play (1) | content (3) | controlling (1) |
|  | cross (2) | inquisitive (1) | awkward (2) | defensiveness (1) |
|  | content (2) | alert (1) | cautious (2) | in pain (1) |
|  | comfortable (2) | emotional (1) | cocky (2) | disappointed (1) |
|  | shy (2) | fed up (1) | discomfort (2) | upset (1) |
|  | tired (2) | ill (1) | stress (2) | expectant (1) |
|  | upset (2) |  | energy (2) | excited (1) |
|  | focused (2) |  | angry (2) | fed up (1) |
|  | watchful (1) |  | amused (2) | frustration (1) |
|  | wary (1) |  | fear (2) | surprise (1) |
|  | aggressive (1) |  | humorous (2) | interest (1) |
|  | quizzical (1) |  | inquisitive (2) | suspicious (1) |
|  | sceptical (1) |  | patient (2) | reluctant (1) |
|  | sorry (1) |  | timid (2) | resentment (1) |
|  | surprise (1) |  | whinnying (2) | serious (1) |
|  | discussing (1) |  | adventurous (1) | pleasure (1) |
|  | diseart (1) |  | apprehensive (1) | pleading (1) |
|  | disciplined (1) |  | asking for  something (1) |  |
|  | exuberant (1) |  | at ease (1) |  |
|  | fear (1) |  | aware (1) |  |
|  | fed up (1) |  | exploration (1) |  |
|  | confident (1) |  | guilty (1) |  |
|  | cool (1) |  | feeling good (1) |  |
|  | cautious (1) |  | focused (1) |  |
|  | chilled (1) |  | hope (1) |  |
|  | unsure (1) |  | hungry/thirsty (1) |  |
|  | like he did something wrong (1) |  | irritated (1) |  |
|  | lonely (1) |  | lacklustre (1) |  |
|  | annoyed (1) |  | nonchalant (1) |  |
|  | apathetic (1) |  | lost (1) |  |
|  | waiting (1) |  | looking for |  |
|  | bouncy (1) |  | mad (1) |  |
|  |  |  | relieved (1) |  |
|  |  |  | restless (1) |  |
|  |  |  | rejected (1) |  |
|  |  |  | shocked (1) |  |
|  |  |  | welcoming (1) |  |
|  |  |  | weighing up a difficult decision (1) |  |
|  |  |  | aggressive (1) |  |
|  |  |  | vane (1) |  |
|  |  |  | proud (1) |  |
|  |  |  | mischievous (1) |  |

Participants used particular words or expressions preferentially for each emotion, but also applied idiosyncratic expressions, i.e. labels that only were used once in the whole human sample. For happiness, the most commonly used words considered accurate, were "excitement", "happiness" and "playfulness"; for positive anticipation were "positive anticipation", "waiting to be given something" and "curiosity"; for fear, "nervousness", "scare" and "anxiety" were the most chosen labels; for frustration, "disappointment", "frustration" and "impatience" were preferred; and finally for the relaxed videos, participants used the labels "neutral", "relaxed" and "no emotion" more frequently. Happiness videos were not mislabelled often, but the more common mislabels used were "peace", "anxiety" and "positive anticipation". According to our approach, these labels would have been correct for relaxed, fear and positive anticipation videos respectively. For positive anticipation stimuli, "excitement", "nervousness" and "neutral" were incorrectly chosen as labels, which would have been considered correctly for happiness, fear and relaxed stimuli, respectively. For the fear videos, "surprise", "tiredness" and "sadness" were the chosen labels; "Surprise" and "sadness" mislabels are different emotional categories not used in this study, while tiredness is not usually considered an emotion. For the frustration videos, participants used mislabels of "happiness", "neutral" and "no emotion", which shows confusion with happiness and relaxed emotion categories. Finally, in the relaxed videos, the more common mislabels were "sadness", "sleepiness" and "tiredness", which indicates that participants were confusing a neutral state with sadness or a non-emotional category of being tired.

**(e) Statistical analysis:**

As research on eye movements in dogs is still in its infancy and it is not known what a typical eye movement pattern in dogs as a species looks like, no outliers were removed from any of the data subsets. There were no obvious data errors and thus the data collected is assumed to include normal variation of spontaneous eye movements in both species. Data exploration was performed with Cleveland plots and boxplots for each data subset, separated by group of interest (e.g. AOI). Normality was verified with histograms with normal curve and Shapiro-Wilk tests. Overdispersion checks were performed by comparing the full model residual variance and the degrees of freedom. Multicollinearity of variables was tested using the VIF function (car R-package).

**Results**

**Body size index:** All data was non-normal, but did not present overdispersion or collinearity between variables. When testing normality of body size for dogs, it was clear that there was a split in the distribution of the body size index for dogs around 2000 units (Fig. S3). Hence, this variable was split into small (<2000) and large (≥2000) body size groups.

**
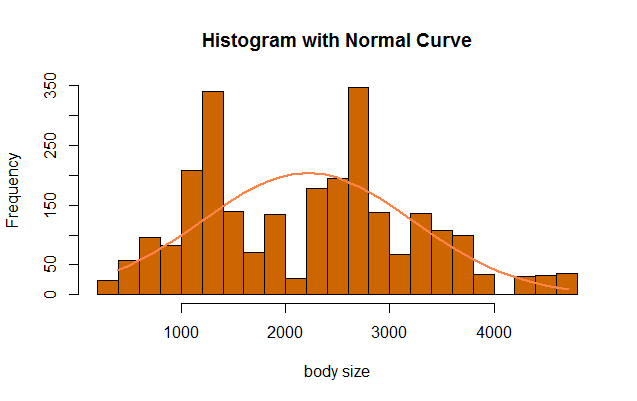
**

**Fig.S3.** Histogram with the distribution of dog body size in our sample, showing a bimodal distribution.

**(a) Human viewers**

**Human viewers modeling for PVT (proportion of viewing time), AOI (area of interest), expression and species viewed:** it was not immediately clear if the best model for the data was the full model or the model with AOI and emotion, since both had very similar AICs (full model AIC: 3365.1 vs model dropping species: 3365.7, ANOVA: F1=2.55, p=.11) and both are significantly much lower than any of the other models. However, by examining the ANOVAs comparing the models, it was clear that the variable species was not impacting the response variable in any way, as whenever it was dropped from the models it gave a non-significant result. It was also non-significant when compared on its own with the null model, strongly suggesting it was not an important variable to explain the PVT variation. Additionally, the AIC rose substantially when emotion was dropped from the best model (best model AIC: 3365.7 vs model dropping emotion: 3452.8, ANOVA: F1=95.1, p=.0001) and it rose even further when AOI was dropped (best model AIC: 3365.7 vs model dropping AOI: 6969.2, ANOVA: F1=3605.5, p=.0001). This confirmed that the best model should include the predictor variables body region (AOI) and emotion viewed, but not species viewed.

**Posthoc Mann-Whitney for head vs body (AOI) per expression:** happiness: *U*=194789, *p*=0.0001, 59%±23 vs 22%±17; positive anticipation: *U*=244604, *p*=0.0001, 72%±24 vs 21%±22; frustration: *U*=255004, *p*=0.0001, 68%±19 vs 20%±15; fear: *U*=249273, *p*=0.0001, 68%±20 vs 22%±16; neutral: *U*=254106, *p*=0.0001, 77%±23 vs 17%±19.

**Human viewers modeling for PVT, age and gender:** The full model was not significantly different from the model with only age, but was significantly different if age was dropped (full model AIC: 4116.6 *vs* model dropping sex: 4114.6, ANOVA: *F1*=0.004, *p*=.95; and vs model dropping age AIC: 4131, ANOVA: *F1*=16.45, *p*=.0001). Additionally, the best model was significantly different from the null model (best model AIC: 4114.6 *vs* null model: 4129.3, ANOVA: *F1*=16.7, *p*=.0001), whereas the model with only sex was not significantly different from the null model (model with sex AIC: 4131 *vs* null model: 4129.3, ANOVA: *F1*=0.30, *p*=.58).

**
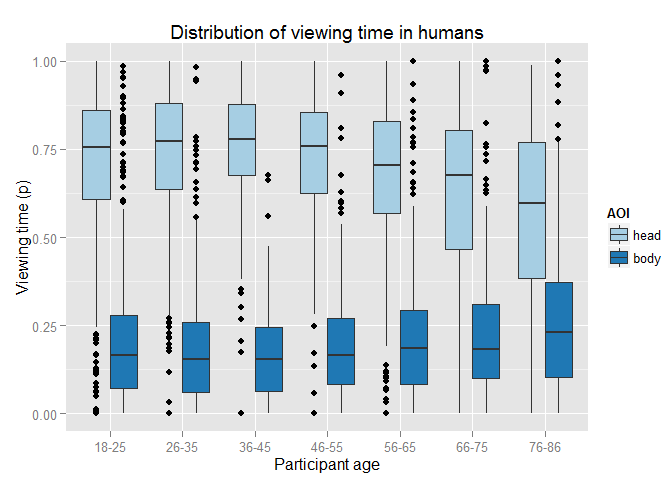
**

**Fig. S4.** Boxplot with distribution of the PVT on the AOIs head and body (of both human and dog stimuli) by human viewers of different age categories. Whiskers represent minimum and maximum, box includes median and interquartile range. Black dots represent outliers.

**
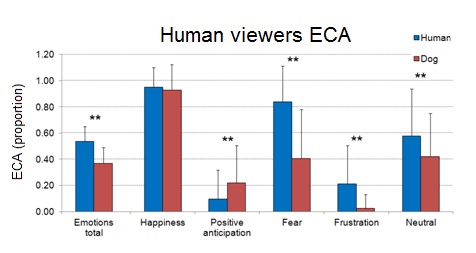
**

**Fig. S5.** Proportion of ECA (Emotion Categorization Accuracy) for human viewers recognizing emotion categories (happiness, positive anticipation, fear, frustration and neutral) in human (blue columns) and dog (red columns) stimuli. Black bars represent Standard Deviation and asterisks represent significant differences between human and dog stimuli (**p<0.01).

**Table S3.** Emotion categorization accuracy (ECA) differences between viewed species (Mann-Whitney, Bonferroni correction p<0.0083).

| **Variables** | **U** | **p (uncorrected)** |
| --- | --- | --- |
| Emotions total | 625796 | 0.0001 |
| Happiness | 40352 | 0.0664 |
| Positive Anticipation | 40352 | 0.0001 |
| Fear | 13128 | 0.0001 |
| Frustration | 21944 | 0.0001 |
| Neutral | 25048 | 0.0001 |

**Table S4.** Emotion categorization accuracy (ECA) differences between viewer's gender (Mann-Whitney, Bonferroni correction p<0.0038).

| **Variables** | **U** | **p (uncorrected)** |
| --- | --- | --- |
| Emotions total | 1508 | 0.3976 |
| Dog emotion | 1599.5 | 0.1655 |
| Human emotion | 1397.5 | 0.8394 |
| Dog happiness | 1533.5 | 0.0843 |
| Dog positive anticipation | 1299 | 0.6630 |
| Dog frustration | 1444.5 | 0.1991 |
| Dog fear | 1366.5 | 0.9875 |
| Dog neutral | 1505 | 0.3587 |
| Human happiness | 1572 | 0.0202 |
| Human positive anticipation | 1272 | 0.4083 |
| Human frustration | 1146.5 | 0.1363 |
| Human fear | 1528.5 | 0.2203 |
| Human neutral | 1437.5 | 0.6415 |

**Table S5.** Emotion Categorization Accuracy (ECA) association with viewer's age (Kendall's Tau, Bonferroni correction p<0.0038):

| **Variables** | **tau** | **z** | **p (uncorrected)** |
| --- | --- | --- | --- |
| Emotion total | 0.0748 | 1.1886 | 0.2346 |
| Dog emotion | 0.0729 | 1.1457 | 0.2519 |
| Human emotion | 0.0388 | 0.5812 | 0.5611 |
| Dog happiness | -0.0989 | -1.3540 | 0.1757 |
| Dog positive anticipation | 0.2907 | 4.0193 | 0.0001 |
| Dog frustration | 0.0742 | 1.0096 | 0.3127 |
| Dog fear | -0.0407 | -0.5837 | 0.5594 |
| Dog neutral | -0.0716 | -1.0169 | 0.3092 |
| Human happiness | -0.0850 | -1.1563 | 0.2475 |
| Human positive anticipation | 0.2094 | 2.8752 | 0.0040 |
| Human frustration | 0.0200 | 0.2773 | 0.7816 |
| Human fear | -0.1140 | -1.5751 | 0.1152 |
| Human neutral | 0.0191 | 0.2732 | 0.7847 |

**(b) Dog viewers**

**Dog viewers modeling for PVT, AOI, expression and species viewed:** The largest rise in the AIC was seen when AOI was dropped from the model (full model AIC: 3241.5 *vs* model dropping AOI: 3320.3, ANOVA: *F1*=80.79, *p*=.0001), which indicated this variable as being the most important for the response variable. Emotion and species were also significant in explaining the data in the model, as when either was dropped, the AIC would significantly rise, with species rising slightly more when dropped from the model (full model AIC: 4116.6 *vs* model dropping emotion: 3258.6, ANOVA: *F1*=25.11, *p*=.0001; and *vs* model dropping species AIC: 3265.5, ANOVA: *F1*=26.02, *p*=.0001).

**Dog viewers modeling for PVT, age and gender:** The models including age, sex, breed, cephalic index and body size as predictor variables had non-convergence issues (even with varied optimizers) probably due to the variable breed not having enough data to estimate the parameters. Hence, breed was dropped from the model and analyzed separately. The AIC of the model only with sex as a predictor variable did not improve significantly when any of the other variables were added, but it significantly increased every time age was dropped from the model (model with age and sex AIC: 3053.8 *vs* model dropping age AIC: 3058.5, ANOVA: *F1*=6.71, *p*=.0096; model with age and cephalic index AIC: 3056.4 *vs* model dropping age AIC: 3060.7, ANOVA: *F1*=6.28, *p*=.012; model with age and size AIC: 3055.4 *vs* model dropping age AIC: 3059.9, ANOVA: *F1*=6.45, *p*=.011). Additionally, the best model also differed significantly from the null model (best model AIC: 3053.6 *vs* null model AIC: 3058.8, ANOVA: *F1*=7.19, *p*=.0073).

**Table S6.** Full non-optimal GLMM model for dog viewers’ PVT as a response variable and the predictor variables age, sex, cephalic index and body size.

| **Predictor factors** | **Estimate** | **SE** | **z** | **p** |
| --- | --- | --- | --- | --- |
| Response factor: PVT | | | | |
| Intercept | -0.70589 | 0.33205 | -2.126 | 0.0335 |
| Age (y) | -0.04114 | 0.01741 | -2.364 | 0.0181 |
| Cephalic index (dolichocephalic) | -0.16128 | 0.15828 | -1.019 | 0.3082  ci_catdolichocephalic -0.16128 0.15828 -1.019 0.3082 |
| Cephalic index (mesaticephalic) | -0.03241 | 0.10717 | -0.302 | 0.7623 |
| Sex (f) | 0.12009 | 0.09059 | 1.326 | 0.1850 |
| Body size (small) | -0.00444 | 0.09520 | -0.047 | 0.9628 |

**Breed analysis:** In order to analyze the effect of breed on PVT, the mixed breed individuals were excluded (N=21) and 71 individuals were entered in the analysis. Due to the low number of individuals for most of the breeds, the 23 breeds were split into the British Kennel Club groups (six groups, as no working breed was present in the sample). There were significant differences between breed groups for overall PVT (Kruskal-Wallis: *H5*=12.50, *p*=.03), but post-hoc Dunn-Bonferroni tests did not reveal any significant differences between groups. Furthermore, no significant differences were found for head or body PVT (Kruskal-Wallis with Bonferroni correction, head: *H5*=7.48, *p*=.19; body: *H5*=9.50, *p*=.09).

**
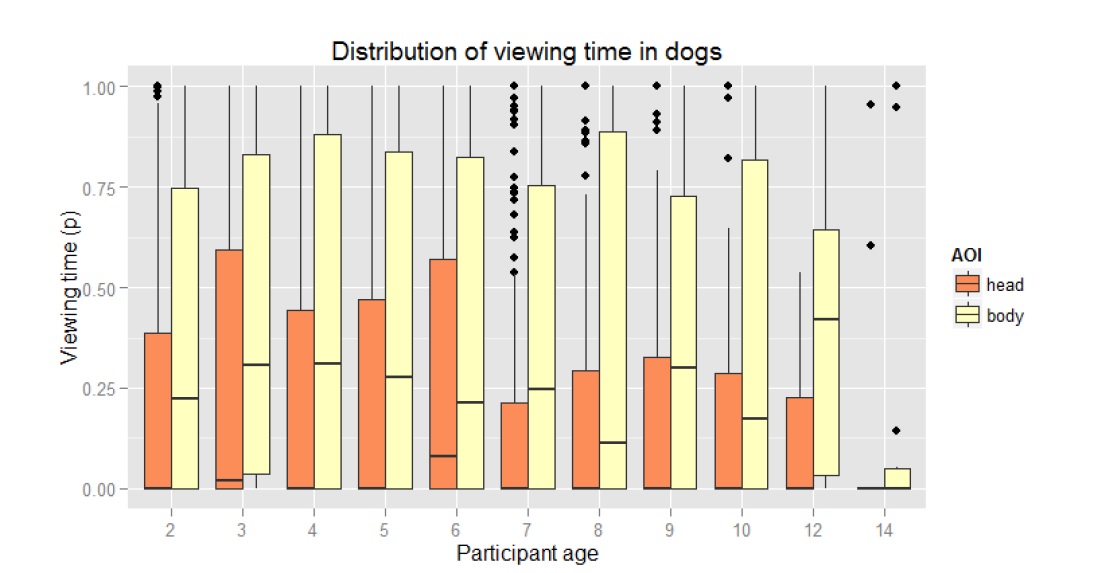
**

**Fig. S6.** Boxplot with distribution of the PVT on the AOIs head and body (of both human and dog stimuli) by dog viewers of different age categories. Whiskers represent minimum and maximum, box includes median and interquartile range. Black dots represent outliers.

**(c) Comparison of human and dog viewers**

**Comparison of human and dog viewers model for PVT, viewers' species, AOI, expression and species viewed:** When comparing the AIC of the models, it was clear that the best model for our data was the full model with the lowest value, which included all predictor variables. The largest rise in the AIC was seen when the variable AOI was dropped from the model, which indicated this variable was the most important for the data (best model AIC: 8519.9 *vs* model dropping AOI AIC: 10301.9, ANOVA: *F1*=1784, *p*=.0001). The second largest change in AIC was seen when dropping the variable participant species from the model (best model AIC: 8519.9 *vs* model dropping participant species AIC: 8714.7, ANOVA: *F1*=196.7, *p*=.0001). The stimulus species and emotion also increased the AIC significantly when dropped, although a smaller increase was seen (best model AIC: 8519.9 *vs* model dropping stimulus species AIC: 8534.7, ANOVA: *F1*=16.7, *p*=.0001; and *vs* model dropping stimulus emotion AIC: 8579.5, ANOVA: *F1*=67.6, *p*=.0001).

**Table S7.** Posthoc tests (Mann-Whitney) of dogs vs humans PVT for AOI, viewed species and expression (Bonferroni correction <0.0023).

| **Variables** | **U** | **p (uncorrected)** |
| --- | --- | --- |
| Viewer species | 4806000 | 0.0001 |
| Head x viewer species | 544070 | 0.0001 |
| Body x viewer species | 1936200 | 0.0001 |
| Head_human_stimuli x viewer species | 108920 | 0.0001 |
| Body_human_stimuli x viewer species | 487400 | 0.0001 |
| Head_dog_stimuli x viewer species | 149230 | 0.0001 |
| Body_dog_stimuli x viewer species | 501750 | 0.3581 |
| Head_human_stimuli_happiness x viewer species | 2747 | 0.0001 |
| Head_human_stimuli_positive anticipation x viewer species | 5469 | 0.0001 |
| Head_human_stimuli_fear x viewer species | 5150 | 0.0001 |
| Head_human_stimuli_frustration x viewer species | 4916 | 0.0001 |
| Head_human_stimuli_neutral x viewer species | 3719.5 | 0.0001 |
| Body_human_stimuli_happiness x viewer species | 19160 | 0.0001 |
| Body_human_stimuli_positive anticipation x viewer species | 23112 | 0.0001 |
| Body_human_stimuli_fear x viewer species | 21063 | 0.0001 |
| Body_human_stimuli_frustration x viewer species | 14038 | 0.4362 |
| Body_human_stimuli_neutral x viewer species | 20210 | 0.0001 |
| Head_dog_stimuli_happiness x viewer species | 5721.5 | 0.0001 |
| Head_dog_stimuli_positive anticipation x viewer species | 6273.5 | 0.0001 |
| Head_dog_stimuli_fear x viewer species | 8360.5 | 0.0001 |
| Head_dog_stimuli_frustration x viewer species | 4382.5 | 0.0001 |
| Head_dog_stimuli_neutral x viewer species | 4388.5 | 0.0001 |
|  |  |  |

**Table S8.** Dogs and humans viewers' PVT Mean and Standard Deviation, by body region (AOI), viewed species and expression:

| **AOI** | **Viewed species** | **Viewed expression** | **Viewer species** | **Mean** | **Standard Deviation** |
| --- | --- | --- | --- | --- | --- |
| Body | Dog | Na | Dog | 0.29 | 0.35 |
|  |  |  | Humans | 0.15 | 0.14 |
|  | Human | Fear | Dog | 0.53 | 0.41 |
|  |  |  | Humans | 0.26 | 0.18 |
|  |  | Frustration | Dog | 0.40 | 0.39 |
|  |  |  | Humans | 0.25 | 0.15 |
|  |  | Happiness | Dog | 0.48 | 0.41 |
|  |  |  | Humans | 0.20 | 0.21 |
|  |  | Neutral | Dog | 0.53 | 0.40 |
|  |  |  | Humans | 0.25 | 0.21 |
|  |  | Positive anticipation | Dog | 0.65 | 0.38 |
|  |  |  | Humans | 0.32 | 0.22 |
| Head | Dog | Fear | Dog | 0.32 | 0.35 |
|  |  |  | Humans | 0.73 | 0.14 |
|  |  | Frustration | Dog | 0.18 | 0.29 |
|  |  |  | Humans | 0.71 | 0.18 |
|  |  | Happiness | Dog | 0.22 | 0.27 |
|  |  |  | Humans | 0.51 | 0.14 |
|  |  | Neutral | Dog | 0.25 | 0.36 |
|  |  |  | Humans | 0.88 | 0.14 |
|  |  | Positive anticipation | Dog | 0.35 | 0.37 |
|  |  |  | Humans | 0.82 | 0.20 |
|  | Human | Fear | Dog | 0.19 | 0.31 |
|  |  |  | Humans | 0.63 | 0.23 |
|  |  | Frustration | Dog | 0.23 | 0.35 |
|  |  |  | Humans | 0.65 | 0.19 |
|  |  | Happiness | Dog | 0.11 | 0.23 |
|  |  |  | Humans | 0.66 | 0.26 |
|  |  | Neutral | Dog | 0.16 | 0.29 |
|  |  |  | Humans | 0.66 | 0.25 |
|  |  | Positive anticipation | Dog | 0.17 | 0.33 |
|  |  |  | Humans | 0.63 | 0.24 |

**Proportion of total viewing time of the stimuli (AOI window):** We report the proportion of total viewing time of the stimuli (AOI window) from human and dog participants in Table S8. Due to species-specific characteristics, the duration of viewed stimuli differs greatly between humans and dogs. The results of a Mann-Whitney test are presented in Table S9, with the test showing significant larger mean proportion of viewing time per video clip for humans (0.83 ± 0.17) when compared to dogs (0.22 ± 0.28). Therefore, all our reported results are standardised by total duration of each video.

**Table S9.** Mean and standard deviation of the proportion of total viewing time of the stimuli (AOI window) for the dog and human subset data.

|  |  | Participant species | | | |
| --- | --- | --- | --- | --- | --- |
| Stimulus species |  | Dog | | Human | |
|  | Stimulus emotion | MEAN | SD | MEAN | SD |
| Human | Happiness | 0.1477 | 0.2447 | 0.8104 | 0.2186 |
|  | Positive anticipation | 0.1756 | 0.2568 | 0.8226 | 0.1730 |
|  | Fear | 0.1694 | 0.2490 | 0.7922 | 0.1989 |
|  | Frustration | 0.1372 | 0.2386 | 0.8228 | 0.1723 |
|  | Neutral | 0.1664 | 0.2517 | 0.8206 | 0.2060 |
| Subtotal |  | 0.1593 | 0.2980 | 0.8137 | 0.1947 |
| Dog | Happiness | 0.3550 | 0.3013 | 0.8173 | 0.1365 |
|  | Positive anticipation | 0.2927 | 0.2997 | 0.8411 | 0.1541 |
|  | Fear | 0.3391 | 0.3170 | 0.8482 | 0.1309 |
|  | Frustration | 0.2313 | 0.2798 | 0.8457 | 0.1685 |
|  | Neutral | 0.2078 | 0.2641 | 0.8623 | 0.1457 |
| Subtotal |  | 0.2852 | 0.2980 | 0.8429 | 0.1492 |
| Total |  | 0.2222 | 0.2811 | 0.8283 | 0.1734 |

**Movie S1 (separate MP4 file).** Video example of human and dog aggregated gaze trace on dynamic full body expressions for species viewed and expression viewed, including one example of happiness, positive anticipation, frustration and fear in dogs, and a neutral example in humans. Each dot represents one viewer, with purple dots from human viewers and green dots from dog viewers.
